# Laparoscopic uncinate process first pancreatoduodenectomy—feasibility study of a modified ‘artery first’ approach to pancreatic head cancer

**DOI:** 10.1007/s00423-017-1597-2

**Published:** 2017-07-11

**Authors:** Michał Pędziwiatr, Magdalena Pisarska, Piotr Małczak, Piotr Major, Mateusz Wierdak, Dorota Radkowiak, Jan Kulawik, Marcin Dembiński, Andrzej Budzyński

**Affiliations:** 1 2nd Department of General Surgery, Department of Endoscopic, Metabolic and Soft Tissue Tumors Surgery, Jagiellonian University Medical College, Kopernika 21, 31-501 Kraków, Poland; 2 Centre for Research, Training and Innovation in Surgery (CERTAIN Surgery), Kraków, Poland

**Keywords:** Pancreatic cancer, Pancreatoduodenectomy, Laparoscopy, Artery first approach

## Abstract

**Purpose:**

The aim of this study was to discuss the feasibility of laparoscopic ‘uncinate first’ pancreatoduodenectomy.

**Methods:**

The analysis included prospectively collected data from 12 consecutive patients undergoing elective pure laparoscopic ‘uncinate process first’ pancreatoduodenectomy (Group 1). They were compared with patients previously operated on with a classical laparoscopic approach (Group 2). The primary outcome was the quality of the resected specimen (lymph node (LN) yield, R0 rate, involved resection margins). Secondary outcomes were perioperative parameters.

**Results:**

The LN yield in Group 1 was 19.3 and in Group 2 it was 13.9 (*p* = 0.03). R0 resection rates did not vary (66.7 vs. 63.2%, *p* = 0.84). Although the involvement of the superior mesenteric artery margin and uncinate process margin seemed lower in Group 1, the difference was not significant. Total operative time (467 vs. 425 min, *p* = 0.13) and resection time (221 vs. 232 min, *p* = 0.34) were similar in both groups. The estimated blood loss in Group 1 was 408 ml, whereas in Group 2 it was 392 ml (*p* = 0.33). Complication rates were 66.7% in Group 1 and 63.2% in Group 2 (*p* = 0.84). Median length of stay was 9 days in both groups (*p* = 0.36). Postoperative complication rates did not differ between groups.

**Conclusions:**

Laparoscopic uncinate first approach is a feasible method for pancreatic head neoplasms. Achieved quality of the specimen is comparable with the traditional laparoscopic approach, whereas intra- and postoperative course is not inferior. However, further studies on larger cohorts are required to fully establish whether the novel approach has potential advantages over classical access in pancreatic head cancer.

## Introduction

The first laparoscopic pancreatoduodenectomy (LPD) was reported over 20 years ago by Gagner and Pomp. Thus far, several comparative studies, published in recent years, have confirmed the feasibility of laparoscopy in cases of pancreatic head malignancy [1, 2]. Although minimally invasive oncologic surgery has become an accepted approach for many abdominal operations, it is still used to a limited extent in pancreatic surgery [3]. It is generally accepted that the laparoscopic approach should follow the same principles as open surgery. Yet, in more difficult cases, a concern arises about the oncologic quality of the operation. Forced attempts to finish the procedure minimally invasively may lead to some compromises in the technique or adjustments to difficult operative conditions and the use of atypical surgical instruments. LPD is, without a doubt, one of the most complex abdominal procedures, involving recognition of difficult anatomy, meticulous vascular dissection and multiple gastrointestinal tract reconstructions. For this reason, the risk of the course of surgery not following the principles of classical pancreatic head surgery is particularly high.

Quite recently, some authors have suggested that the so-called artery first approach (meaning superior mesenteric artery (SMA) dissection in the early phase of resection, before any irreversible step is taken) has potential advantages, such as early determination of resectability and decreased R1 resection rate [4–6]. So far, this approach has been described mostly in open pancreatoduodenectomy. The data on artery first LPD are sparse [7]. Therefore, the aim of this paper was to discuss the feasibility of laparoscopic ‘uncinate process first’ pancreatoduodenectomy.

## Methods

### Setting

All procedures were performed in a university tertiary referral unit, mostly involved in elective surgical treatment of abdominal oncologic diseases. Starting from December 2015, we have changed our approach (both in open and laparoscopic access) to uncinate process first, where the dissection of the SMA is performed at the very beginning of the procedure. Any case of intraoperative difficulties with dissection or uncertainty regarding tumour infiltration is converted to open surgery. The annual volume of our institution is 70–75 patients undergoing pancreatoduodenectomy for various indications and 30 of them are eligible for laparoscopy. All specimens were assessed by one of two experienced pathologists according to the standardized protocol proposed by Verbeke et al. [8]. R1 resection margin was defined when it was close or <1 mm according to Esposito et al. [9].

### Patients

**Fig. 1:**
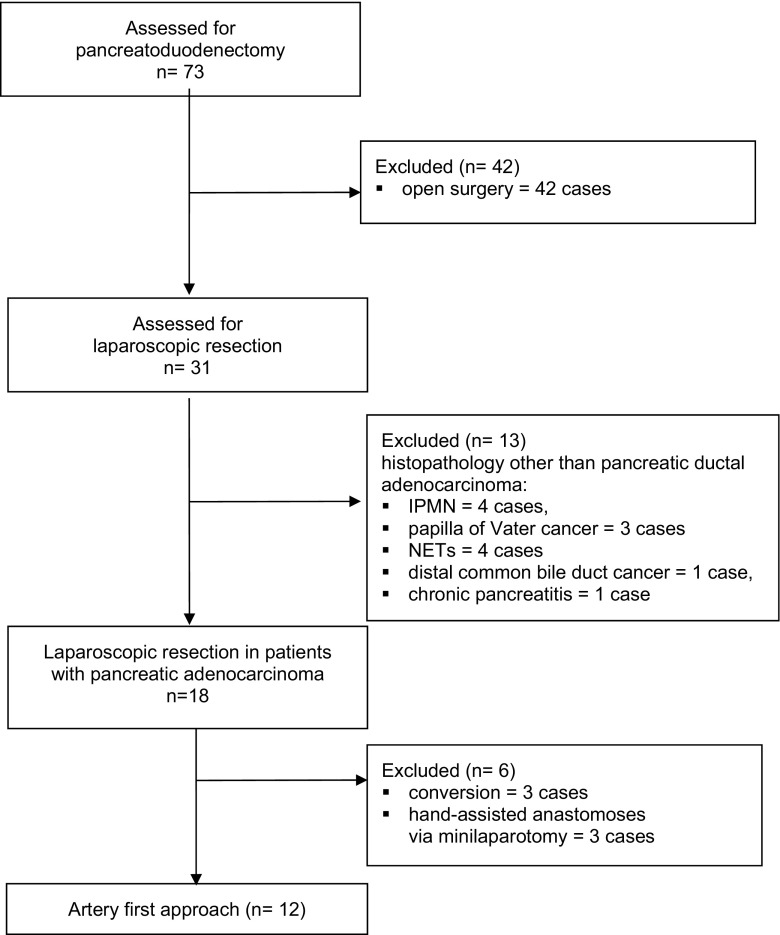
Patient flowchart

The study included prospectively collected data from 12 consecutive patients undergoing elective laparoscopic uncinate process first pancreatoduodenectomy for pancreatic ductal adenocarcinoma between December 2015 and December 2016. All laparoscopic procedures were performed by the same surgeon (AB), with extensive expertise in laparoscopic hepatobiliary surgery. The uncinate process first was compared with 19 patients with pancreatic ductal adenocarcinoma previously operated on with the classical laparoscopic approach over the period of 12 months before the artery first approach was introduced. Patients with histopathology other than pancreatic ductal adenocarcinoma or hand-assisted/converted cases were excluded from the final analysis. Moreover, patients with suspected vascular infiltration or those undergoing preoperative chemotherapy are not submitted to laparoscopic dissection in our institution. Figure 1 shows the patients’ flow through the study.

### Measured outcomes

Our primary outcome was the quality of resected specimen (lymph node (LN) yield, R0 rate, involved resection margins—posterior surface of the uncinate process, pancreatic neck margin, superior mesenteric artery margin, superior mesenteric vein margin). All specimens are assessed according to the Verbeke protocol [8]. They are inked according to an agreed colour code to facilitate specimen orientation. Secondary outcomes were operative and postoperative parameters (total operative and resection time, blood loss, complication rate during hospital stay and within 30 days postdischarge).

### Operative technique

**Fig. 2:**
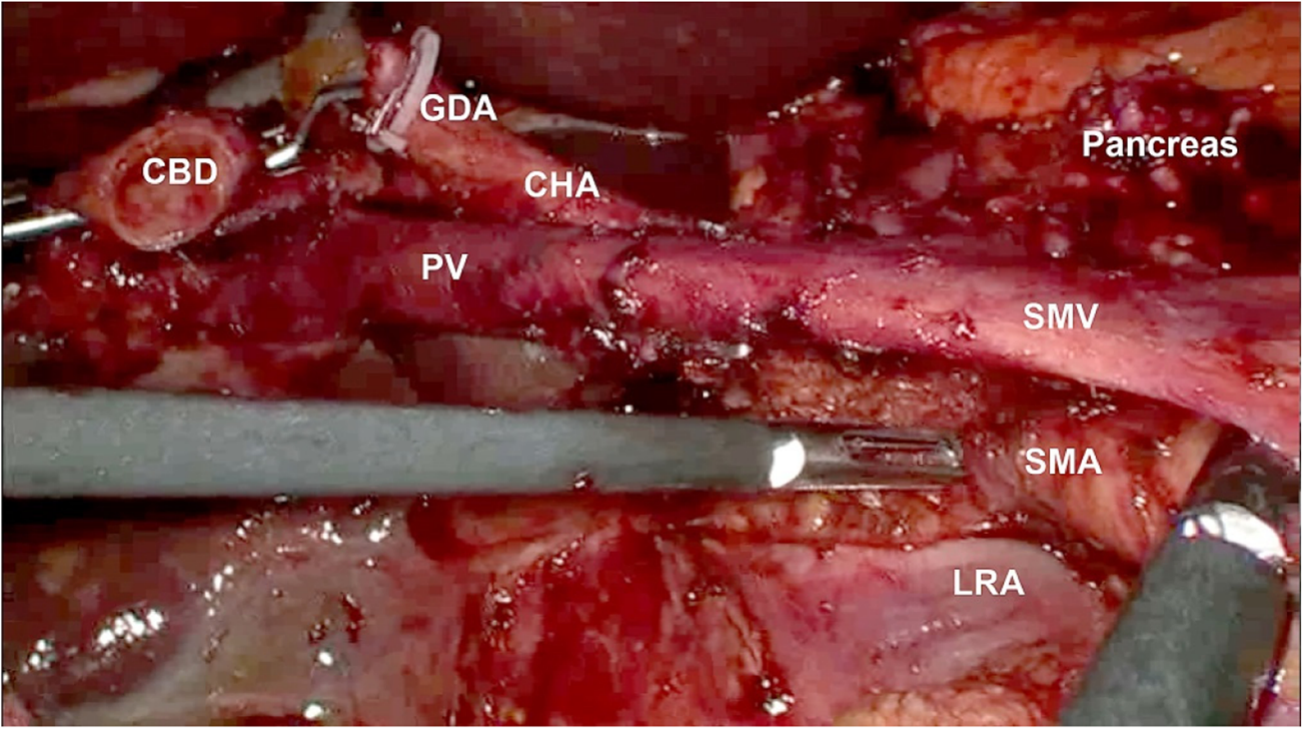
Operative field after resection part

The right flexure of the colon is fully mobilized and a modified Kocher manoeuvre is performed with a wide mobilization of the duodenum and the head of the pancreas from the retroperitoneal adhesions (with partial resection of the prerenal fascia and full exposition of anterior aspect of the vena cava and left renal vein). In the uncinate process first, the SMA is identified at the early stage of the procedure. The dissection is carried out along the aorta until the origin of the SMA is identified according to the technique described by Hackert et al. [10]. In the next step, the superior mesenteric vein (SMV) is being exposed below the pancreas. Traction of the duodenum and the head of the pancreas towards the anterior abdominal wall and rotation of the small bowel mesentery expose the infrapancreatic segment of the SMA. The dissection then follows the course of the artery towards its origin at the aorta. Once the resectability is confirmed, common hepatic and proper hepatic arteries and the common bile duct are identified. The gastroduodenal and right gastric arteries are clipped and divided. The first portion of the duodenum and the first jejunal loop are transected with Echelon® stapler. The head of the pancreas, with the tumour, is dissected from the superior mesenteric vessels (starting from the SMA followed by the SMV and portal vein). The larger arterial and venous branches, including the inferior panreaticoduodenal artery, are clipped and cut off. The neck of the pancreas is divided as the last step of the resection phase. Bleeding from the cut surface of the organ is controlled with sutures. Electrocautery is not routinely used to avoid damage of the pancreatic parenchyma. Additional lymphadenectomy of Group 8 (located around the common hepatic artery), Group 9 (around the celiac trunk) and Group 12 (located around the hepatic proper artery) lymph nodes is performed. Figure 2 shows the operative field after resection.

In the ‘classical approach’, the SMA is not identified at the beginning of the dissection phase. The neck of the pancreas is dissected from the SMV and portal vein confluence. It is then transected and the venous plane is followed, so that the uncinate process and the head of the pancreas are freed from surrounding tissues. The SMA plane is not identified.

All anastomoses are performed laparoscopically, regardless of the approach. Drains near pancreaticojejunostomy are left in most patients, while drains near hepaticojejunostomy are not placed routinely.

### Statistical analysis and ethical approval

All data were analysed with Statsoft STATISTICA v.12. The results are presented as mean ± standard deviation (SD). The study of categorical variables used the chi-square test of independence. The Shapiro-Wilk test was used to check for normal distribution of data and the T-student test was used for normally distributed quantitative data. For non-normally distributed quantitative variables, the Mann-Whitney *U* test was used. Results were considered statistically significant when *p* value was found to be less than 0.05.

The study was approved by the local Ethics Review Committee. All procedures were performed in accordance with the ethical standards laid down in the 1964 Declaration of Helsinki and its later amendments. Written informed consent was obtained from all patients before surgery.

## Results

Patients in Group 1 were comparable to patients in Group 2 in regard to demographic parameters, except for ASA grade (Table 1).

**Table 1:** Demographic characteristics of patient groups

Parameter	Group 1 (uncinate process first approach)	Group 2 (classical approach)	*p* value
Number of patients, *n*	12	19	–
Females, *n* (%)	6 (50%)	7 (36.8%)	0.47
Males, *n* (%)	6 (50%)	12 (63.2%)	
Mean age, years ± SD (median)	58.0 ± 12.4 (56)	62.3 ± 8.5 (62)	0.09
BMI, kg/m^2^ ± SD (median)	24.5 ± 3.1 (24.5)	25.6 ± 2.8 (25.6)	0.38
ASA 2, *n* (%)	12 (100%)	13 (68.4%)	0.01
ASA 3, *n* (%)	–	6 (31.6%)	
Any comorbidity	8 (66.7%)	10 (52.6%)	0.44
Cardiovascular	4 (33.3%)	4 (21.1%)	0.45
Hypertension	2 (16.7%)	8 (42.1%)	0.12
Diabetes	2 (16.7%)	3 (15.8%)	0.95
Pulmonary disease	2 (16.7%)	1 (5.3%)	0.30

There were no significant differences in the stage of cancer between groups. The LN yield in Group 1 was 19.3 and in Group 2 it was 13.9 (*p* = 0.03). R0 resection rates did not differ (66.7 vs. 63.2%, *p* = 0.84). The involved margins are presented in Table 2. Although involvement of the superior mesenteric artery margin and uncinate process margin seemed lower in Group 1, we did not perform the statistical analysis due to small sample sizes.

**Table 2:** Pathologic results

Parameter	Group 1 (uncinate process first approach)	Group 2 (classical approach)	*p* value
AJCC			0.78
AJCC II A, *n* (%)	7 (58.3%)	9 (47.4%)	
AJCC II B, *n* (%)	3 (25.0%)	7 (36.8%)	
AJCC III, *n* (%)	2 (16.7%)	3 (15.8%)	
T category			0.93
pT2	3 (25.0%)	5 (26.3%)	
pT3	9 (75.0%)	14 (73.7%)	
N category			0.55
pN0	7 (58.3%)	9 (47.4%)	
pN1	5 (41.7%)	10 (52.6%)	
Lymph nodes, *n* (%)	19.3 ± 8.2 (16)	13.9 ± 9.4 (13)	0.03
Resection			0.84
Resection R0, *n* (%)	8 (66.7%)	12 (63.2%)	
Resection R1, *n* (%)	4 (33.3%)	7 (36.8%)	
Resection margin involvement			
SMAM	2	4	
SMVM	1	1	
PUPM	2	4	
PNM	–	–	
BDM	–	–	

R1 resection margin was defined when it was close or <1 mm according to Esposito et al.[9]

*SMAM* superior mesenteric artery margin, *SMVM* superior mesenteric vein margin, *PUPM* posterior surface of the uncinate process margin, *PNM* pancreatic neck margin, *BDM* bile duct margin

Total operative time (467 vs. 425 min, *p* = 0.13) as well as resection time (221 vs. 232 min, *p* = 0.34) were similar in both groups. The estimated blood loss in Group 1 was 408 ml, while in Group 2 it was 392 ml (*p* = 0.33). Complication rates were 66.7 and 63.2%, respectively (*p* = 0.84). Median length of stay was 9 days in both groups (*p* = 0.36) (Tables 3 and 4).

**Table 3:** Postoperative characteristics of patient groups

Parameter	Group 1 (uncinate process first approach)	Group 2 (classical approach)	*p* value
Mean total operative time, min. ± SD (median)	466.7 ± 53.8 (445)	425.0 ± 85.1 (420)	0.13
Mean resection time, min. ± SD (median)	220.7 ± 47.8 (230)	232.3 ± 51.8 (245)	0.34
Mean intraoperative blood loss, ml ± SD (median)	408.3 ± 166.3 (300)	391.7 ± 180.7 (250)	0.33
Median length of hospital stay (IQR)	9 (8–12)	9 (6–12)	0.36
Patients with complications, *n* (%)	8 (66.7%)	12 (63.2%)	0.84
Clavien-Dindo 1, *n* (%)	2 (16.7%)	4 (21.1%)	0.34
Clavien-Dindo 2, *n* (%)	4 (33.3%)	7 (36.8%)	
Clavien-Dindo 3, *n* (%)	2 (16.7%)	–	
Clavien-Dindo 5, *n* (%)	–	1 (5.3%)	
Readmission, *n* (%)	–	1 (5.3%)	-

**Table 4:** Types of complications according to Clavien-Dindo classification

		Group 1 (uncinate process first approach)	Group 2 (classical approach)
I	Chyle leak	1	1
	Pancreatic fistula grade A	1	2
	Surgical site infection	–	1
II	Urinary tract infection	–	1
	Delayed gastric emptying (requiring TPN)	3	4
	Pancreatic fistula grade B	1	1
	Surgical site infection (requiring antibiotics)	–	1
III	Biliary anastomotic leakage (reoperation)	1	–
	Postoperative bleeding (reoperation)	1	–
V	Death (anastomotic leakage, massive bleeding)	–	1

## Discussion

In this study, we have confirmed the feasibility of laparoscopic uncinate process first approach for pancreatic head malignancy. The change in the operative technique did not have any negative influence on the operative time, blood loss and complications. In addition, pathologic specimen quality was comparable.

Currently, there is a lot of evidence that laparoscopic surgery can be successfully implemented in most of gastrointestinal cancer cases leading to reduced postoperative morbidity without compromising long-term survival. It is also significant that, nowadays, patients prefer minimally invasive access for various reasons [11–13]. However, the surgeons’ acceptance for laparoscopic surgery in pancreatic head malignancy is still low. This is due to the limited number of studies showing the clinical advantages of laparoscopy over open surgery [3]. LPD still remains an extremely difficult operation, with a long learning curve and prolonged operative time [14].

Only recently, a modified artery first approach was described in pancreatic head surgery, which allows early determination of SMA involvement [15]. There are several potential advantages of the artery first approach. They include the following: better resection of mesopancreas with a more adequate lymphadenectomy, reduced blood loss and an easier identification of aberrant right hepatic artery [16]. Although nowadays, triphasic computed tomography or endoscopic ultrasound allows for the delineation of resectable, borderline resectable or non-resectable tumours, they may be insufficient in selected cases. Therefore, the use of laparoscopy for early assessment of SMA infiltration may have potential benefits, one of them being a better selection of candidates for vascular resection or patients who would benefit from neoadjuvant chemotherapy and a second-look operation. Moreover, faster recovery and better general status after exploratory laparoscopy may allow introducing chemotherapy earlier.

The number of resected lymph nodes in our series is comparable with that of previous reports in both laparoscopic and open surgery [17, 18]. Although there is a difference in the number of harvested lymph nodes between groups, we are not certain whether this is due to the change in the operative approach. This aspect has to be confirmed in a larger trial comparing the classical approach with the artery first approach. In addition, there are more accurate prognostic factors related to lymph nodes, such as the number of positive nodes or lymph node ratio [19]. In addition, there are no advantages of extended lymphadenectomy on survival [20]. Moreover, pancreatic cancer spread is characterized not only by lymphatic metastases, but also by perineural invasion that may potentially lead to lymphatic spread of cancer [21]. Therefore, a simple lymphadenectomy, without the resection of peripancreatic soft tissues and extrapancreatic nerve plexus, is considered oncologically insufficient. For this reason, meticulous skeletonization of the mesenteric vessels, with regional lymphadenectomy and perivascular neural and soft tissue removal, is highly recommended [22, 23]. According to some authors, the artery first approach is superior over the classical in terms of better mesopancreas dissection [15]. This term was first used by Gockel et al. in 2007 [24]. Mesopancreas does not contain any surrounding fascia and it is defined as an anatomical space bounded by the pancreatic neck (anteriorly), pancreaticoduodenal fascia (posteriorly) and superior mesenteric vessels (medially). It does, however, contain lymph nodes, nerves and smaller vessels [25]. It has been suggested that novel approach to mesenteric vessels may facilitate mesopancreas resection, thus lowering the incidence of R1 resection [21]. Although this sounds logical, it still has to be investigated with well-designed trials to fully answer the question whether it lowers the recurrence rate and survival. Besides, there are no standardized protocols of mesopancreas assessment as those used in rectal cancer surgery, for instance.

According to the review by Sanjay et al., there are six different approaches to SMA that may be considered as the artery first approach [15]. In our series, we adapted the so-called uncinate process first approach described in open surgery by Hackert et al. [10]. In this technique, the resection is performed in a retrograde way starting from the jejunum, whereas the transection of the pancreas is the last step of the resection phase. We agree with Hackert’s observations that in this way it is possible to safely and completely dissect the uncinate process from the retroperitoneum and the superior mesenteric vein under visual control of the vein and the artery.

In our material, we did not find any differences in R0/R1 rates. The R1 rate in our group is relatively high, which is due to fact we use the classification proposed by Esposito et al. (R1 resection margin is defined as positive margin within <1 mm of the tumour) [9]. Lack of difference in R0 rates, on the one hand, confirms that the artery first approach enables comparable quality of resection. On the other hand, we must admit that both groups were relatively small, therefore we interpret these results cautiously.

The laparoscopic uncinate process first approach allowed us to achieve comparable, if not better quality of the specimen. Another important observation from our study was that it was not associated with a worse intraoperative and perioperative course. The resection time, intraoperative blood loss and postoperative complications were not different between groups.

However, one of the greatest limitations of the study is that the learning curve in the artery first approach has not been completed yet. Although most parts of the procedure do not differ regardless of the used approach and practically only one particular step is different from what we have learnt, this may still introduce bias. Besides, this single-centre analysis includes relatively small groups of patients. Nevertheless, we clearly showed the feasibility of this technique in laparoscopic setting.

## Conclusion

In conclusion, we can confirm that the laparoscopic uncinate first approach is feasible and all steps of the procedure can be successfully applied to laparoscopic access. The artery first approach helps to identify early resectability. Whether it would increase negative margins of resection, improve disease-free survival and increase survival are yet unknown. More studies, including larger cohorts of patients, are required to fully establish whether the novel approach has potential advantages over the classical access to pancreatic head malignancy.
